# A meta-analysis of the association between post-traumatic stress disorder and cancer risk

**DOI:** 10.3389/fpsyt.2023.1281606

**Published:** 2023-10-30

**Authors:** Juanjuan Yang, Wei Jiang

**Affiliations:** ^1^Department of Health Management, The Second Affiliated Hospital of Xi’an Jiaotong University, Xi’an, Shaanxi, China; ^2^Department of Oncology, The Second Affiliated Hospital of Xi’ an Jiaotong University, Xi’an, Shaanxi, China

**Keywords:** PTSD, stress, cancer incidence, ovarian cancer, breast cancer

## Abstract

**Background:**

Several studies have investigated the link between post-traumatic stress disorder (PTSD) and cancer risk but reported mixed results. The objective of our study was to investigate the association between PTSD and cancer risk.

**Methods:**

Studies published in English about the relationship between PTSD and cancer incidence were systematically searched. We performed a meta-analysis to estimate the relative risks (RR) and 95% confidence intervals (CI) for cancer incidence.

**Result:**

A total of 3,129 articles were screened. Finally, 8 articles and 11 studies were included in the meta-analysis. We found that PTSD was not associated with cancer risk compared with controls. For site-specific cancer, our results showed that women with PTSD were associated with higher risk of ovarian cancer than controls. However, PTSD was not associated with the risk of gastrointestinal cancer, breast cancer and lung cancer.

**Conclusion:**

These analyzes based on studies published in English suggest that PTSD is associated with ovarian cancer risk, although the evidence base is very limited. Future studies are needed to investigate the mechanisms that PTSD diagnosis influenced cancer incidence depending on types of cancer.

## 1. Introduction

Post-traumatic stress disorder (PTSD) is one of the most common stress-related mental disorders and caused by exposing to a severe stressful event (1). During stressful events, the hypothalamic–pituitary–adrenal axis (HPA) and sympathetic nervous system are mobilized, which can influence the occurrence and development of diseases by releasing stress hormones, such as norepinephrine, epinephrine and glucocorticoids (2). On one hand, cellular and molecular studies have indicated that stress hormones affected tumor biology by stimulating oncogene activation, modulating programmed cell death, inhibiting DNA damage repair and stimulating tumor angiogenesis (3). On the other hand, previous researches revealed a close association between PTSD and immune system, which could result in a higher risk for developing autoimmune and inflammatory disorders, such as cancer, cardiovascular disease, atherosclerosis and diabetes (4, 5). The immune dysfunction has influence on tumor behaviors. Our previous studies also indicated that chronic stress promoted tumor growth by regulating immune function (6, 7).

Epidemiological studies showed that stress-related mental disorders play a crucial role in the tumor incidence and tumor progression (8, 9). For example, the cancer incidence of patients with depression and anxiety was higher than that of the general population, besides, the cancer-specific mortality (CSM) and all-cause mortality (ACM) in cancer patients with depression and anxiety were higher than those of the general cancer patients (8, 10). However, the association between PTSD and cancer risk has been investigated but has remained unclear.

A previous epidemiological study found that adjusted postwar mortality for cancer was associated with PTSD among Vietnam Theater veterans (11). However, some cross-sectional studies reported that PTSD was associated with cancers diagnosed in life (12), while another cross-sectional study reported that PTSD was not associated with cancer incidence (13). Moreover, the cross-sectional study was only able to describe the association between PTSD and cancer incidence but unable to draw conclusions on the causal relationship. Therefore, the cancer incidence risk in patients with PTSD remains uncertain.

Based on the biological and behavioral mechanisms that may explain how stress-related mental disorders affect cancer incidence rate, we hypothesize that there may be a positive correlation between PTSD and cancer incidence. Several cohort studies and case–control studies have investigated the association between PTSD and cancer incidence but have reported mixed results (14–18). A meta-analysis to pool the conflicting results is missing. Herein, we performed a meta-analysis s to investigate the association between PTSD and cancer risk.

## 2. Methods and materials

### 2.1. Inclusion and exclusion criteria

The inclusion criteria including: 1) studies investigating the relationship between PTSD and cancer incidence; 2) PTSD patients were diagnosed according to well-validated diagnostic criteria; 3) articles published in English language before Aug 4, 2023. We excluded studies without control group. Studies were also excluded if PTSD was diagnosed later than cancer. JW and YJ independently conducted research choices based on inclusion and exclusion criteria, with differences were resolved through consensus.

### 2.2. Search strategy

Literature search was conducted by YJ and JW through the PubMed, Web of Science and Embase online databases. Literature searches by title or abstract were updated on Aug 4, 2023 and were restricted to English written full-text articles. The following search terms were used: (“posttraumatic stress disorder” OR “PTSD” OR “post-traumatic stress disorder”) AND (“cancer” OR “malignancy” OR “sarcoma” OR “carcinoma” OR “neoplasm” OR “tumor” OR “tumor”). The database’s of our research strategy is title or abstract. We also searched the references of the identified relevant studies to collect any additional studies that met the inclusion criteria. Our study was completed according to the Preferred Reporting Items for Systematic Reviews and Meta-Analyzes (PRISMA) guidelines (19).

### 2.3. Quality assessment

The Newcastle Ottawa Scale (NOS) was used to evaluate the quality of the included study. Conduct quality assessment on eight projects in three fields: selection (4 projects, 1 star each), comparability (1 project, up to 2 stars), and exposure (3 projects, 1 star each) (20). Studies including at least 7 stars to be considered good, those at least 4 stars to be considered fair, and those including less than 3 stars to be considered poor.

### 2.4. Data extraction and data analysis

YJ and JW independently extracted data [year of publication, sample size, measurement of PTSD, first author’s name, cancer type, adjusted confounders, and association strength of cancer incidence with corresponding 95% confidence intervals (CI)] and discussed any discordance to reach agreement.

### 2.5. Statistical analysis

Data was analyzed through Stata 14.0 (Stata Corp LP, College Station, TX, United States). We evaluate heterogeneity between studies using Q test and I^2^ statistic. An I^2^ ≥ 50% was considered highly heterogeneous, and we combined the results using a random effects model. The relative risk (RR) and its 95% CI were used to evaluate the effect size. If RR is not reported, we assume that the risk ratio (HR) or odds ratio (OR) could be considered approximately equivalent to RR (10). The significance level was defined as *p* < 0.05. Publication bias was examined using Egger’s test. In order to resolve heterogeneity, we also performed subgroups analyzes based on cancer types and study designs.

## 3. Results

### 3.1. Search results

**Figure 1:**
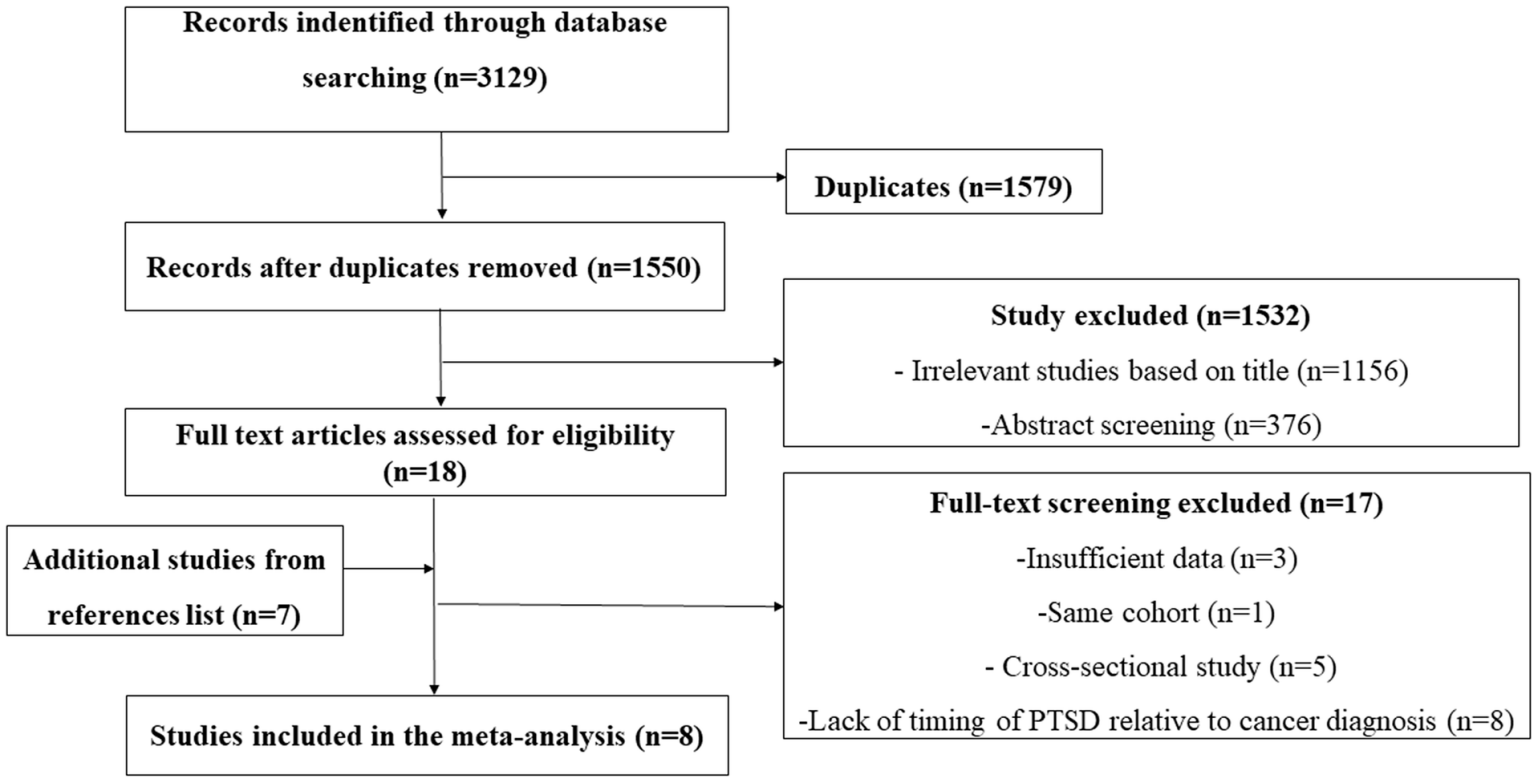
Study selection flowchart.

We searched 3,129 articles and we excluded 1,579 articles because of duplicate results. Among the remaining 1,550 articles, we excluded 1,156 irrelevant studies based on title through. We excluded 376 through abstract screening because studies did not report the relationship between PTSD and cancer incidence, case report, reviews, studies research PTSD symptoms in cancer patients. Finally leaving 18 records for further full-text screening. Another 7 records were identified from the references of the studies. We excluded 17 articles for the following reasons insufficient data, same cohort, cross-sectional study and lack of timing of PTSD relative to cancer diagnosis as shown in Figure 1. Finally, 8 articles s were included in our study (Figure 1) (14–18, 21–23).

### 3.2. Study characteristics and quality evaluation

The detailed study characteristics including first author’s name, sample size, measurement of PTSD, cancer type, year of publication, and adjusted confounders are shown in Table 1. Of these, five studies reported all types of cancer incidence risk (14, 15, 21–23), two studies reported the incidence risk of ovarian cancer (15, 16), three studies reported breast cancer incidence risk (15, 17, 18), two studies reported the incidence risk of gastrointestinal cancer (15, 18), two studies reported the incidence risk of lung cancer (15, 18). The Newcastle-Ottawa Scale values of included studies ranging from 6 to 8quality, which included the quality of studies was good (Table 1). Overall, the studies were published between 2014 and 2022.

**Table 1:** Characteristics of included studies for cancer risk of patients with PTSD.

First author, publication year	Sample size (*N*)	Measure of PTSD	Design	Cancer type	Adjusted confounders	NOS score
Britvić, 2015	1,326	ICD-10, DSM-IV	Case–control	All	Age, education, marital status and employment status	7
Cohn, 2018	93,616	ICD-10	Case–control	Lung cancer	Smoking status, obesity, antidepressant use	7
Cohn, 2018	108,701	ICD-10	Case–control	Colorectal cancer	Smoking status, obesity, antidepressant use	7
Cohn, 2018	154,637	ICD-10	Case–control	Breast cancer	Smoking status, obesity, antidepressant use	7
Cohn, 2018	133,152	ICD-10	Case–control	Prostate cancer	Smoking status, obesity, antidepressant use	7
Gradus, 2015	4,131	ICD-10	Cohort	All	Sex, substance abuse diagnoses, age at PTSD diagnosis, induction time	7
O’Neill, 2014	52,095	DSM-IV	Cohort	All	Age, sex, person-year, country, mental disorder	6
Roberts, 2019	54,710	DSM-IV	Cohort	Ovarian cancer	Age, history of tubal ligation, parity, drugs use, smoking, BMI, physical activity, family history of breast and ovarian cancer	8
Sommer, 2022	2,941	DSM-IV	Cohort	All	Age, gender, education, income, ethnicity, marital status, military characteristics, mental health condition, number of types of traumas	7
Tian, 2022	167,836	ICD	Cohort	All	Age, sex, education, income, marital status, Charlson comorbidity index, substance use disorders	7
Vin-Raviv, 2014	265	DSM-IV	Case–control	Breast cancer	Age, individual hunger score, education, BMI, self-perceived hunger score, alcohol consumption, number of traumatic events	7

ICD, International Classification of Diseases; DSM, Diagnostic and Statistical Manual of Mental Disorders; NOS, Newcastle-Ottawa Scale; PTSD, Posttraumatic Stress Disorder; BMI, Body Mass Index.

### 3.3. Associations between PTSD and cancer risk

**Figure 2:**
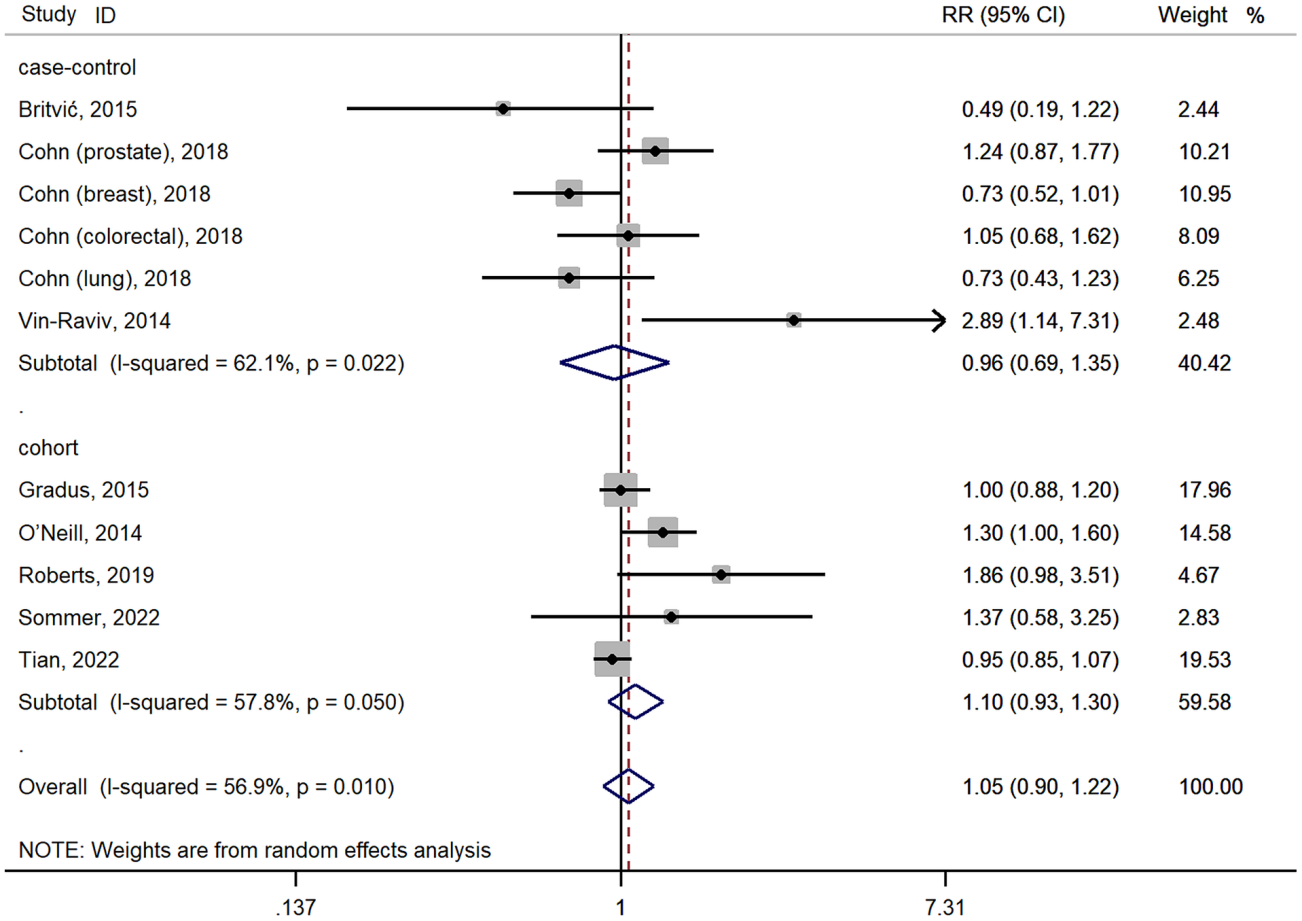
Forest plots for subgroups analysis of the association between PTSD and cancer risk based on study designs. PTSD, posttraumatic stress disorder; CI, confidence interval; RR, relative risks.

**Figure 3:**
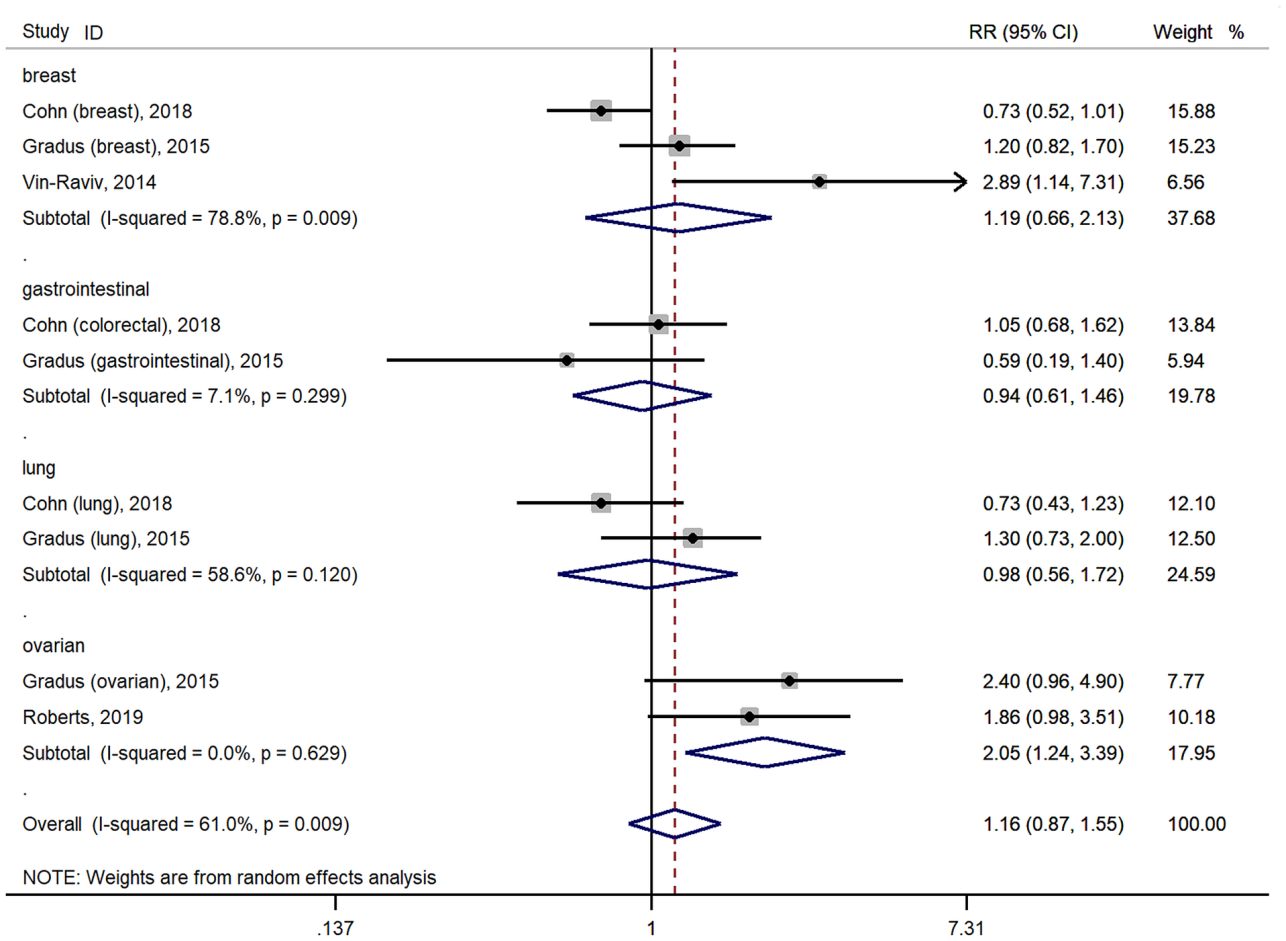
Forest plots for subgroups analysis of the association between PTSD and cancer risk based on cancer types. PTSD, posttraumatic stress disorder; CI, confidence interval; RR, relative risks.

The meta-analysis results of 11 studies showed that PTSD was not associated with all cancer risk from random-effects model (RR: 1.05, 95% CI: 0.90–1.22; Figure 2), with a high heterogeneity (I^2^ = 56.9%; *p* = 0.01). The result of Egger’s test showed there was no publication bias among included studies (*p* = 0.427). As cohort studies and case–control studies included in our study. We also performed subgroups analyzes based on study designs to resolve heterogeneity. However, subgroups analyzes based on study designs did not resolve heterogeneity (Figure 2). We also performed subgroups analyzes based on cancer types. For site-specific cancer, subgroups analyzes were performed based on cancer types showed that PTSD was associated with a significantly higher risk of ovarian cancer incidence (*n* = 2; RR: 2.05, 95% CI: 1.24–3.39; Figure 3), with a low heterogeneity (I^2^ = 0%; *p* = 0.629). However, 3 studies yielded a pooled RR of 1.19 (95% CI: 0.66–2.13; Figure 3) for the association between PTSD and breast cancer risk based with a High heterogeneity (I^2^ = 78.8%, *p* = 0.009). Similarly, the subgroups meta-analysis results showed that PTSD was not associated with risk of gastrointestinal cancer (*n* = 2; RR: 0.94, 95% CI: 0.61–1.46; I^2^ = 7.1%; Figure 3) and lung cancer (*n* = 2; RR: 0.98, 95% CI: 0.56–1.72, I^2^ = 58.6%; Figure 3) incidence.

## 4. Discussion

Our results showed that PTSD patients were significantly more vulnerable to develop ovarian cancer. There was no relationship between PTSD and cancer incidence of breast, gastrointestinal and lung. It is the first meta-analysis to investigate the link between PTSD and cancer incidence.

Stress-related mental health disorders have close relationship with cancer incidence. Kaster and colleagues reported that PTSD was associated with increased risks of all cancer, gastrointestinal cancer but not breast cancer (12). However, some recently epidemiological studies reported that PTSD was not associated with increased risks of cancer (21–23). Roberts and colleagues reported that women with high PTSD symptoms were significantly more vulnerable to develop ovarian cancer than women with no trauma exposure but this association attenuated to not statistically significant after adjustment for health and ovarian cancer risk factors (16). A lead cause of PTSD in women is sexual assault. Women with PTSD from sexual assault may be less likely to seek preventative care for the sex organs may contribute to high risk of ovarian cancer (24). Our results showed that PTSD was associated with elevated risk of ovarian cancer incidence but did not increase incidence risks of breast cancer, gastrointestinal cancer and lung cancer. Although our meta-analysis only included 8 articles and 11 studies for analysis, more cohort studies are needed to confirm our results. Our current study provides future directions for elucidating the potential associations between PTSD and cancer risk. Despite there were the limited studies to investigate the associations and between PTSD disorders and site-specific cancer risk, our study indicated that the impact of PTSD on cancer incidence may depend on cancer types, and more experimental studies are needed to explore the underlying mechanisms.

As for the underlying mechanisms of association between PTSD and cancer risk, many factors may be involved. PTSD is one of the most common stress-related mental disorders and caused by exposing to a severe stressful event (1). PTSD affects tumor biology through stress responses. Stress activates sympathetic nervous system and HPA, with consequent presence of higher levels of neurotransmitters, such as epinephrine, glucocorticoid, norepinephrine (25, 26). Subsequently, epinephrine or norepinephrine regulate tumor tumorigenesis through modulating programmed cell death, stimulating oncogene activation, inhibiting DNA damage repair and stimulating tumor angiogenesis (3, 27, 28). PTSD can also destruct immunity through stress hormones. Chronic stress exerts a negative influence on immune function through neurotransmitters such as epinephrine and norepinephrine (29). The proportion of immune cells and levels of inflammatory factors in peripheral blood of people exposed to a severe stress were different from those of the general population (5, 30). However, cancer is one of the immune associated diseases and immune dysfunction influences tumor behaviors. Suppression of immune responses and elevation of inflammatory markers could increase potential for tumorigenesis (31). Finally, unhealthy lifestyle and behavioral differences may indirectly contribute to the cancer incidence. Individuals with stress tend to have unhealthy lifestyles, such as smoking and alcohol misuse, which may increase the risk of cancer (22, 32). Our study found that PTSD is associated with ovarian cancer risk, but the evidence base is very limited. More experimental studies are needed to investigate the exact the association and mechanism between PTSD and cancer incidence.

On one hand, a cancer diagnosis can be a stressful event that can lead PTSD (33, 34). For example, nearly 30 % of patients with acute myeloid leukemia reported clinically significant PTSD symptoms 1 month after initiating intensive chemotherapy (33). On the other hand, clinical study found that PTSD was associated with increased mortality among cancer patients while treatment of PTSD was associated with improved cancer-related outcomes (9). Therefore, more studies are needed to determine the bidirectional associations between PTSD and cancer diagnosis.

Our study has some important clinical implications. First, our results showed that PTSD in women was associated with a significantly increased risk of ovarian cancer compared with controls. However, our results also reported that there was no association between PTSD and cancer incidence of breast, gastrointestinal and lung. Our findings indicated that the association between PTSD and cancer risk might be complicated and depended on the cancer types. Second, the results of our meta-analysis highlight that women with PTSD deserve focused care for ovarian cancer screening and treatment. Early screening and effective intervention of ovarian cancer for female PTSD patients have clinical and public health importance to prevent and treat cancer. Finally, basic studies indicated that antipsychotics could inhibit cancer proliferation, induce apoptosis and suppress metastasis in a variety of cancer cells *in vitro* and *in vivo* models (35). In addition, chronic stress promotes ovarian tumor progression by increasing IL-6 expression through stimulation activates Src tyrosine kinase (36). Therefore, the optimal treatment recommendations for ovarian cancer patients with PTSD should be jointly made by oncologists and clinical psychiatrists.

Our study has several limitations. First, significant heterogeneity was recorded for studies on cancer incidence. We performed subgroups analyzes based on cancer types and study designs to resolve heterogeneity. Subgroups analyzes based on study designs did not resolve heterogeneity while subgroups analyzes based on cancer types resolved heterogeneity of ovarian cancer and gastrointestinal cancer. The source of heterogeneity could not be fully analyzed because we only included 8 articles and 11 studies for analysis. We considered it justified to pool the studies because the value of I^2^ is 56.9% and we used a random effects model. Second, animal studies indicated that stress could promote cancer incidence while antipsychotics could inhibit cancer incidence (37, 38). Cohn’s study adjusted the confounder of antidepressant use (18). We could not exclude the confounding factors, which may confound the association between PTSD and cancer risk, such as the use of antipsychotics, occupational information, eating habits, body mass index, and smoking. Therefore, more research is needed to clarify the relationship between PTSD and cancer risk. Thirdly, although a comprehensive search for literatures published in English was conducted in PubMed, Web of Science, and Embase, we did not include literature found in other databases such as PTSDpubs, not written in English literatures, unpublished data or literatures published as conference abstracts. Moreover, we failed to indicate a certain association between PTSD and cancer incidence. Finally, although meta-analyzes are informative, it is rather preliminary to add only two or three studies, especially when the RR is still quite high, which means there may have been lack of power in these studies. Thus, our conclusion is weak and need further research to confirm.

## 5. Conclusion

In summary, this meta-analysis based on studies published in English provides evidence that PTSD is associated with ovarian cancer risk while PTSD was not associated with breast cancer, gastrointestinal cancer and lung cancer risk. Future studies are needed to investigate the mechanisms that PTSD diagnosis influenced cancer incidence depending on types of cancer.

## Data availability statement

The raw data supporting the conclusions of this article will be made available by the authors, without undue reservation.

## Author contributions

JY: Data curation, Formal analysis, Writing – original draft, Writing – review & editing. WJ: Data curation, Formal analysis, Funding acquisition, Writing – original draft, Writing – review & editing.
